# Antisecretory Factor in rat cerebellar granule cells augments inhibitory current via GABA_A_ receptors: a mechanism for vertigo reduction in Menière’s disease?

**DOI:** 10.1007/s00249-026-01837-4

**Published:** 2026-03-24

**Authors:** Aroldo Cupello, Virginia Bazzurro, Alberto Diaspro, Elena Gatta, Kliment Gatzinsky, Eva Jennische, Stefan Lange, Mauro Robello, Elena Angeli

**Affiliations:** 1 Department of Physics, University of Genoa, Genova, Italy; 2 Nanoscopy and NIC@IIT, CHT, Istituto Italiano di Tecnologia, Genova, Italy; 3 Department of Neurosurgery, Sahlgrenska University Hospital, Gothenburg, Sweden; 4 Department of Medical Biochemistry and Cell Biology, Institute of Biomedicine, Sahlgrenska Academy, University of Gothenburg, Gothenburg, Sweden; 5 Department of Infectious Diseases, Institute of Biomedicine, University of Gothenburg, Gothenburg, Sweden; 6 Department of Clinical Microbiology, Region Västra Götland, Sahlgrenska University Hospital, Gothenburg, Sweden

**Keywords:** Menière’s disease, Vertigo, Cerebellar granule cells, GABA, Anti-secretory Factor

## Abstract

In this review we propose a mechanism by which the antisecretory factor AF may decrease vertigo symptoms in Menière’s disease patients. This review is based on our previously published work and suggests a way of interpretation of the results obtained in view of the clinical effect of AF factor against vertigo caused by Menière’s disease. The Antisecretory Factor (AF) is an endogenous protein regulating water and ion transport across cellular membranes. AF was originally discovered and isolated from rat and pig pituitary gland but later it has been isolated from all mammalian and non-mammalian tissues examined. It has shown positive effects in patients with Mb Crohn, Colitis Ulcerosa and Mastitis with regression of clinical symptoms and inflammatory parameters. AF is also effective in suppressing traumatically induced increases of intra-cranial pressure (ICP). Another interesting activity of this protein is its anti-vertigo action in patients with Menière’s disease. We studied rat cerebellar granule cells in culture and demonstrated increases by AF of the inhibitory current activated by GABA at synapses. This implies strong inhibition of cerebellar granule cells in situ. The connection of cerebellum to other brain areas would finally result in activation of vestibular Deiters’ neurons in the brainstem, controlling antigravity neurons in the spinal cord. These actions may contribute to counteracting vertigo in Menière’s disease.

**Graphical abstract:**

**Figure:**
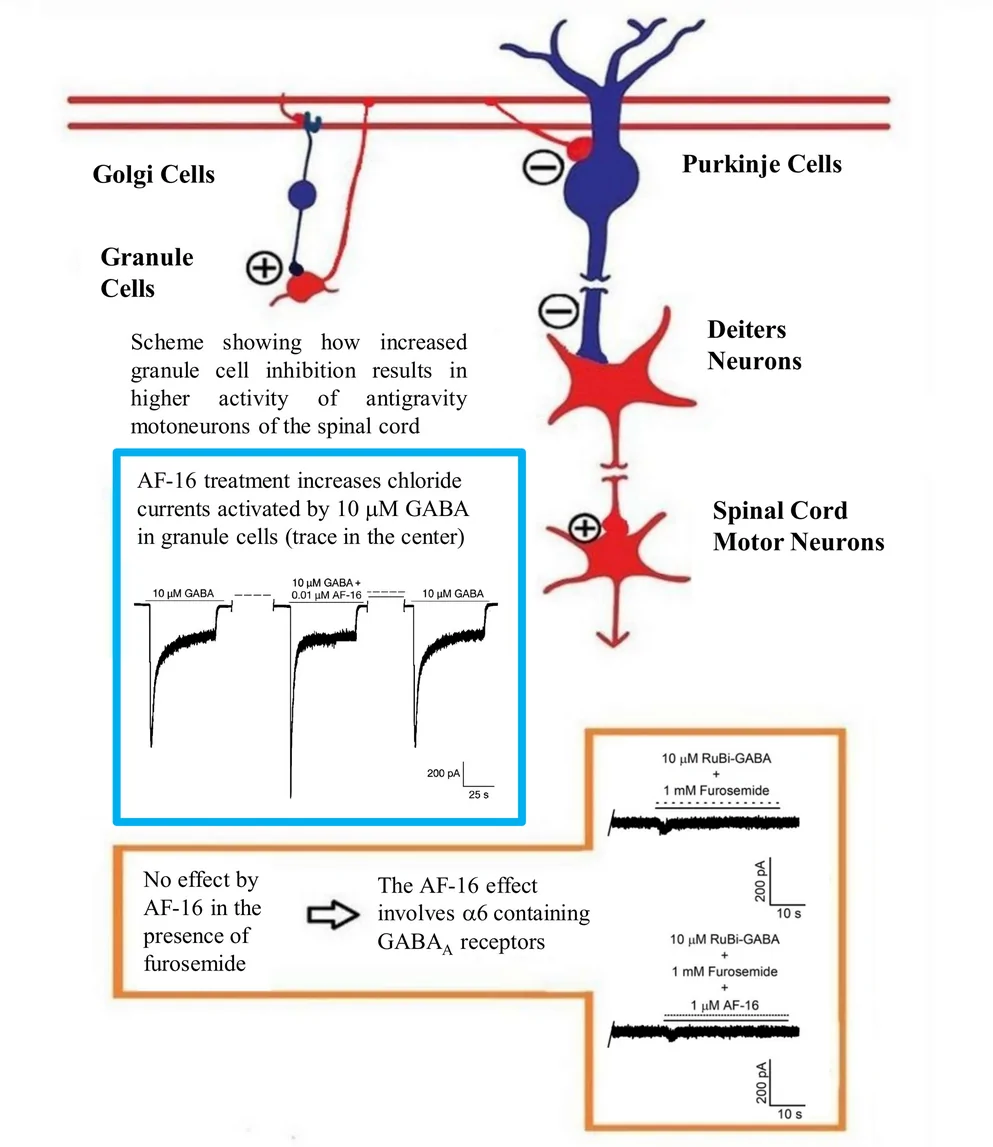

## Antisecretory Factor – biologic characteristics

The Antisecretory Factor (AF) is a 378 amino acids long, endogenous peptide chain characterized in detail (Lonnroth and Lange 1986; Lonnroth et al. 1988; Johansson et al. 1995; Lange and Lonnroth 2001). The AF protein was originally isolated from tissues obtained from the rat or pig pituitary gland and/or the intestinal mucosa. AF functions in vivo as a part of the innate immune system, and serves in vivo/in vitro as a regulator of water and ions transport across cellular membranes (Davidson and Hickey 2004a, b).

All tissues so far examined both in rat and pig demonstrate the presence AF protein. The biologically active part of the protein mediating anti-secretory/anti-inflammatory effects *in vivo/in vitro*, emanates from the amino terminal part of the full length protein chain, and the sequence is located between the amino acid no. 35 and no. 42 and consist of IVCHSKTR (Johansson et al. 1997). A 16 amino acids long peptide AF-16, containing the biologically active part, has been found more stable and is used for experimental work (Al-Olama et al. 2011, 2015; Dzebo et al. 2014).

The major part of AF present in a healthy human displays a biologically inactive form of the AF protein which can be transformed into AF activity if stimulated by i.e. diarrhoea, and/or inflammation. The active form of AF will adjust a deranged cellular transport back to normal values (Lonnroth et al. 1988).

Endogenous AF can also be stimulated to activity by intake of food and drinks (Bjorck et al. 2000). Specially processed cereals, called SPC-Flakes, stimulate the production of active AF in the human body (Laurenius et al. 2003; Hanner et al. 2004). The switch of this factor from its inactive to active form is clearly demonstrated after SPC flakes intake, by ELISA testing of blood samples in a rat model, although the exact mechanism of its activation is not definitely determined (Johansson et al. 2011). A preformed, active form of AF can also be administered directly to the patient via oral intake of egg-yolk preparations, derived from yolk with a high content of AF (Zaman et al. 2014, 2018). Such yolk is prepared by feeding the egg-lying hens for 6–8 weeks with a composition of cereals that stimulates to increased AF synthesis/activation, reflected by a high content of AF in the egg yolk (Lange et al. 1994; Kaya et al. 2017). Such yolk is processed by a “spray-drying process” and is designated as Salovum.

SPC-Flakes and Salovum have been classified by the Swedish Medical Products Agency and The Medical board of EU as “Food for specific medical purposes” (EU directive 1999/21/EG).

The results achieved so far suggest that AF peptides should preferentially be tested in vivo in situations in which the main cause of the disease is a deranged water and ions transport, such as, obviously, cholera or Menière’s disease, which is presumably due to excessive production of endolymph in the inner ear.

## Antisecretory Factor - action in clinical studies

Clinical studies of the AF-stimulating capacity of SPC-Flakes, or intake of preformed AF via Salovum will be used as part of the experimental tools. The peptide AF-16 is presently not allowed for clinical use in humans. The ability of AF to regulate secretory processes and affect inflammation has so far led to significant, clinical effects on diarrhea (Laurenius et al. 2003; Zaman et al. 2014, 2018), inflammatory bowel diseases (Bjorck et al. 2000; Eriksson et al. 2003) and mastitis (Svensson et al. 2004). All of these diseases present a severe inflammation in various tissues, often in combination with a deranged water transport.

## Central nervous system effects

Experimental studies have shown that rats subjected to traumatic brain injury develop a high intracranial pressure (ICP), which can be suppressed by intravenous/intranasal administration of AF-16 (Al-Olama et al. 2015). Patients subjected to a traumatic brain injury often develop an edema induced, high ICP, refractory to conventional medical treatment. However, administration of Salovum to patients can reduce increased ICP when used as a complement to conventional intensive care, as demonstrated in two different pilot studies (Gatzinsky et al. 2020; Cederberg et al. 2020). This specific Salovum effect appears to be selective for trauma-induced brain edema, since no suppressing ICP effect is registered for glioma-induced ICP increase (Carstam et al. 2025). The best and not stressful ways for Salovum administration appear to be the intravenous or intranasal ones. Thus, clinical work is ongoing in order to transform the peptide AF-16 to become a classified drug for intravenous/intranasal use, primarily aimed for suppression treatment of trauma-induced high ICP. A Phase I test for the AF-16 peptide was performed and approved by the Swedish Medical Products Agency during the autumn 2022. This Phase I test is now followed by an ongoing Phase IIA.

The pathogenesis of Menière’s disease is unclear, but disturbances in the secretory processes in the inner ear have been suggested. AF treatment should therefore tentatively be useful, and AF treatment in clinical practice for Menière’s disease has provided optimistic results, especially when it comes to reducing the vertigo part of the disease (Hanner et al. 2003, 2004, 2010; Scarpa et al. 2020; Viola et al. 2020). However, an improvement of the hearing losses seems to be more difficult to achieve.

## Cerebellum circuitry, inhibition of granule cells by GABA_A_ receptors

**Fig. 1:**
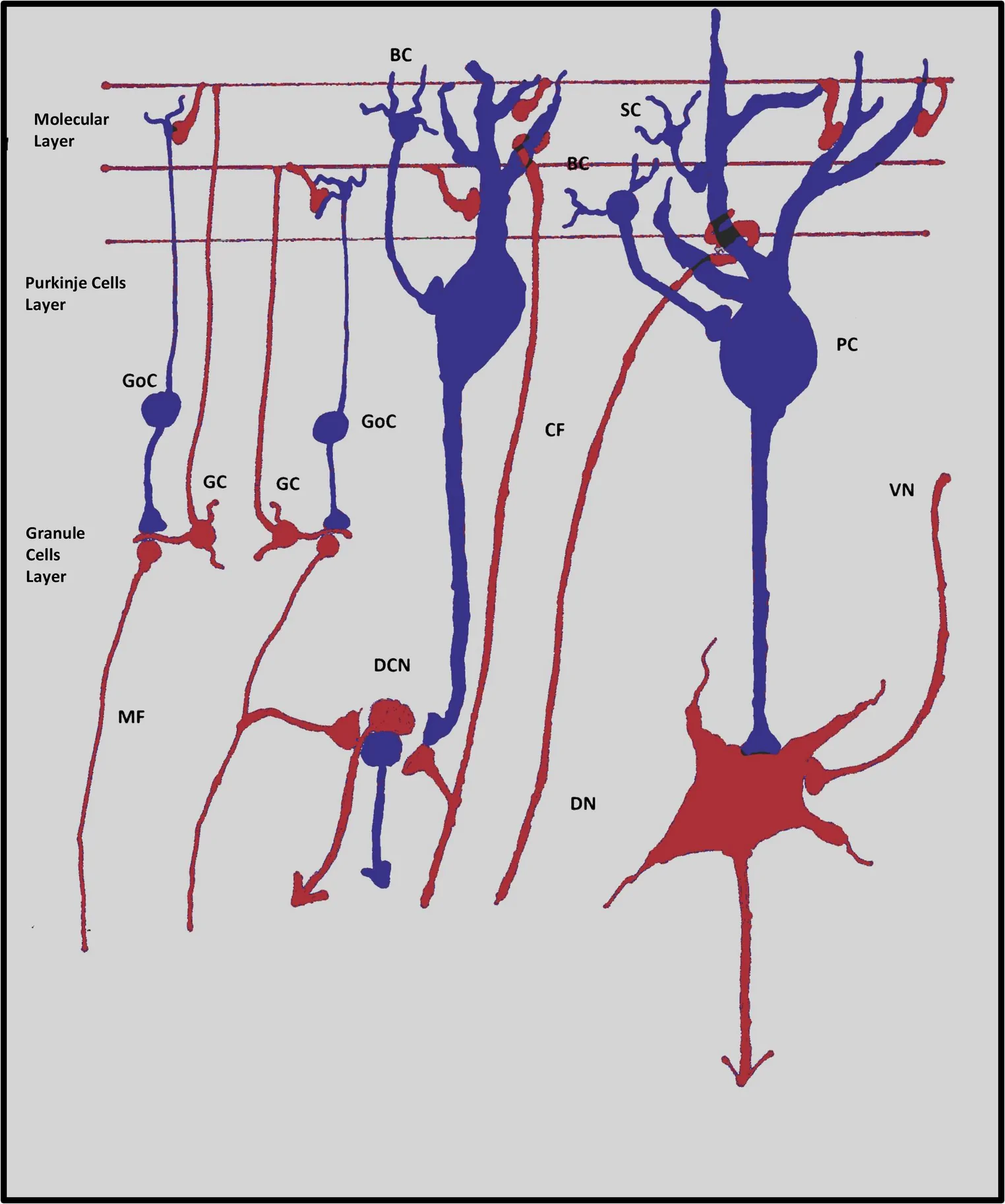
Schematic representation of the circuitry of the cerebellar cortex. Excitatory glutamatergic neurons are in red, inhibitory GABAergic neurons are in blue. BC: Basket Cell; CF: Climbing Fibers; DCN: Deep Cerebellar Nuclei; DN: Deiters Neuron; GC: Granule Cell; GOC: Golgi Cell; MF: Mossy Fibers; PC: Purkinje Cell; SC: Stellate Cell; VN: Vestibular Nerve

The mammalian cerebellum is mainly involved in the regulation of motor activity via its circuitries and connections, which integrate physiological messages arriving by the glutamatergic inputs of the mossy and the climbing fibers (Ito 2006). The first type of connection terminates in the glomeruli in the cerebellar cortex, where it contacts dendrites of the granule cells. The granule cells, in turn, send processes in the molecular layer. These processes form the parallel fibers, which develop contact via excitatory glutamatergic terminals with the dendrites of the Purkinje cells. In the molecular layer, the parallel fibers also contact inhibitory interneurons (stellate and basket cells) which, in turn, regulate the activity of the Purkinje cells (Fig. 1). The Purkinje cells are involved in sending neural messages out of the cerebellum, either indirectly via their inhibitory GABA mediated inputs regulating the activity of deep cerebellar nuclei (DCN) or directly inhibiting mono-synaptically vestibular Deiters’ neurons of the lateral vestibular nucleus (LVN), via cerebello-vestibular projections (Ito and Yoshida 1966; Ito et al. 1966; Ito 2006; D’Angelo 2018). The cerebello-vestibular projection keeps the giant vestibular Deiters’ neurons under continuous inhibitory GABA mediated control preventing, under normal conditions, their powerful activation of spinal cord neurons involved in anti-gravity activity (Ito 2006). This activation must come into play in case of need to maintain the posture equilibrium of the body, when it is challenged by the gravity force.

Within the cerebellar circuits, an important role is played by the GABAergic inhibitory brake on granule cells. Golgi cells, are activated by incoming mossy fibers after these fibers elicit excitatory postsynaptic potentials and action potentials in granule cells by glutamate release at glomeruli (Sieghart 2024). They provide a GABA-ergic inhibitory brake to granule cells. The GABA receptors involved are mainly of the GABA_A_ types, chloride channels that are opened by interaction of GABA with its recognition site. In particular, in the cerebellum, at variance with other brain regions, these receptors mainly contain the α6 subunit (Sieghart et al. 2022). The receptor types involved are different whether engaged in direct synaptic fast or in tonic inhibitory current. In the first case α_n_β_2/3_γ_2_ types are active whereas in the latter one α_n_β_2/3_δ types enter into action. From a functional point of view, the first ones provide a rapid regulation of the incoming activation of the granule cells and of the cerebellum, the second ones act as a sort of a “filter” by limiting the amount of information entering the cerebellum (Farrant and Nusser 2005; Sieghart 2024). The synaptic GABA_A_ receptors can be of the α_1_β_2/3_γ_2_, the α_1_α_6_β_2/3_γ_2_, or of the α_6_β_2/3_γ_2_ subtypes whereas the receptor at the basis of tonic inhibition are of the α_1_β_2/3_δ, the α_1_α_6_β_2/3_δ, or of the α_6_β_2/3_δ subtypes (Sieghart et al. 2022).

**Fig. 2:**
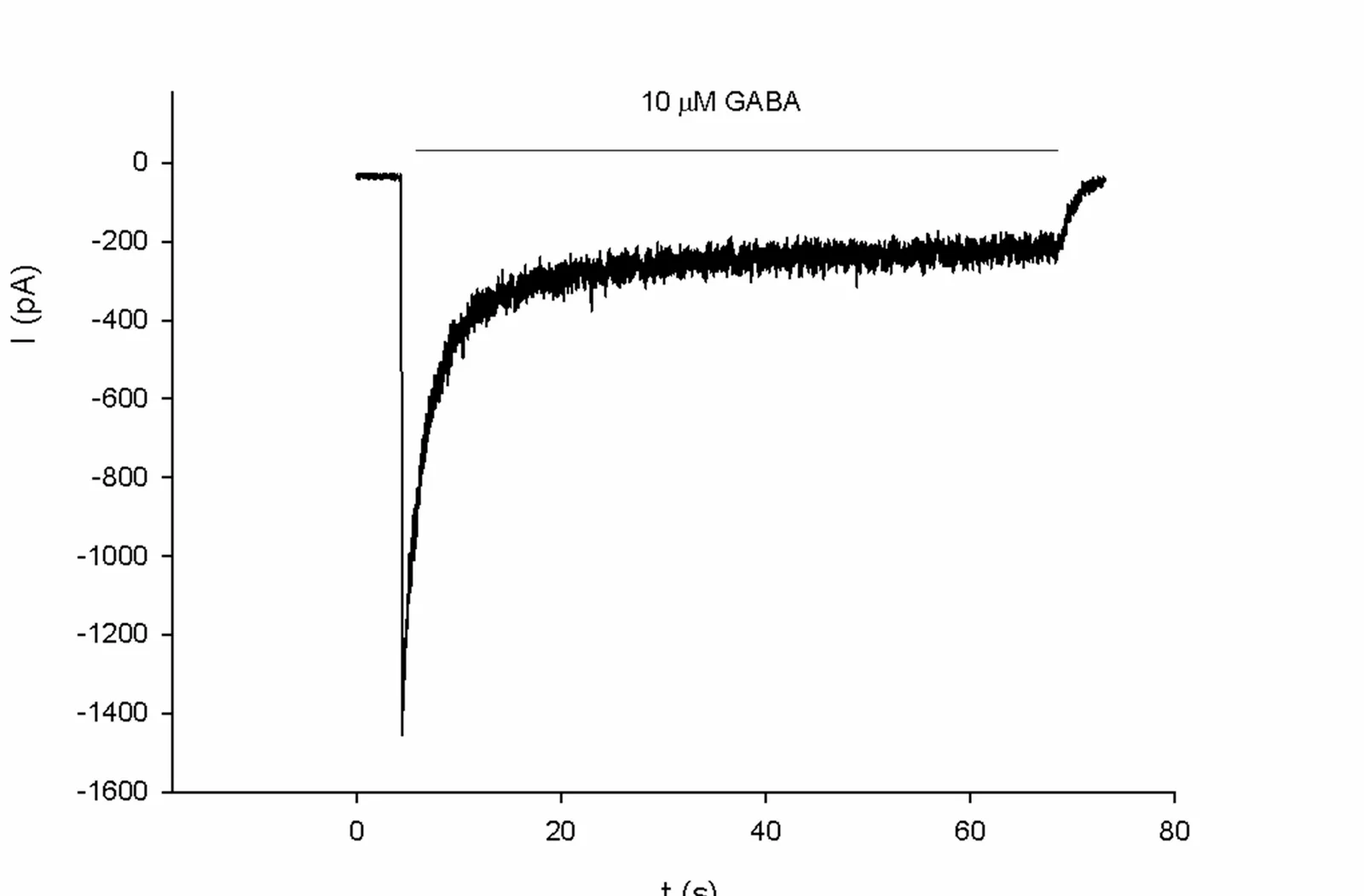
Chloride current activated by 10 µM GABA in cerebellar granule cells (whole cell patch clamp). The registration displays a first rapidly activating and desensitizing component (“peak”), followed by a “steady state” component

Our group has been investigating the biochemistry, physiology and pharmacology of GABA_A_ receptors of rat cerebellar granule cells in primary culture starting from the early 1990’s (Robello et al. 1993). By the whole cell patch-clamp technique it was found that GABA activates chloride currents having a peak and a steady state (Fig. 2). The steady state component presents a higher affinity for GABA (Hill constant K 3.7 µM for the steady state vs. 7.5 µM for the peak component) and, at variance from the peak, it is not potentiated by agonists at the benzodiazepine site (Baldelli et al. 1994). The peak component is partially activated by flunitrazepam (Baldelli et al. 1994) and it is mainly due to the α_1_β_2/3_γ_2_, α_1_α_6_β_2/3_γ_2_ and α_6_β_2/3_γ_2_ subtypes of GABA_A_ receptors, whereas the steady state one involves the α_1_β_2/3_δ subtype (Gatta et al. 2009). The component we describe as “peak” corresponds in situ to the “phasic” fast synaptic one, the one we describe as “steady state” corresponds in situ to tonic inhibition due to extracellular GABA (Farrant and Nusser 2005).

## AF and cerebellar granule cells GABA_A_ receptors activity

In this section we summarize previously published experiments showing the effects of AF on GABA_A_ receptors of the rat cerebellar granule cells.

**Fig. 3:**
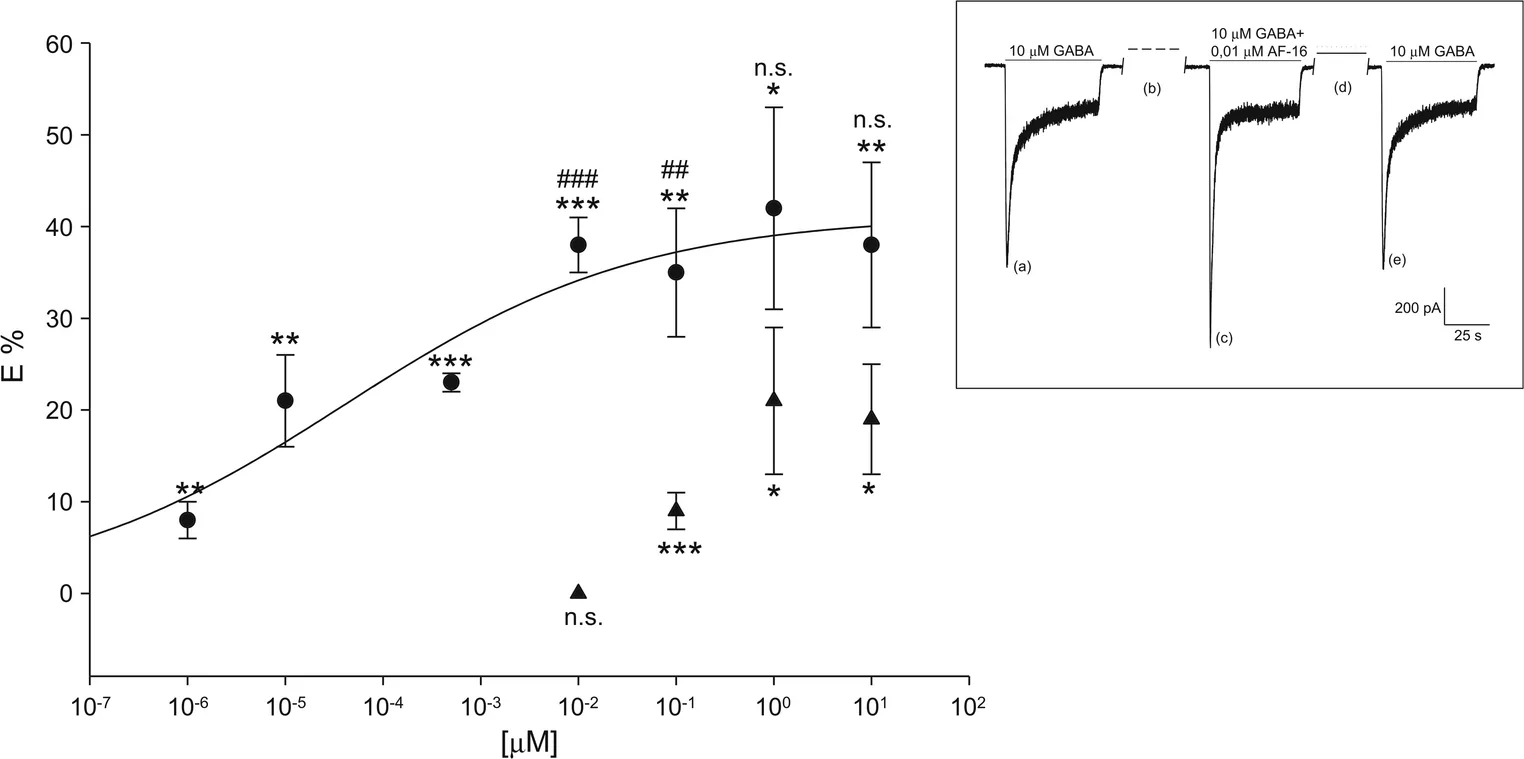
Relative increase (E %) of the *peak chloride currents* activated by 10 µM GABA after 3 min perfusion of increasing concentration of AF-16 (closed circles). The closed triangles represent 30 s of AF-16 perfusion. Vertical bars represent SEMs. Number of experiments was at least three in all cases. Statistical significance of E% increase is reported as asterisks: * *p* < 0.05; ** *p* < 0.01; *** *p* < 0.001. To the statistical significance of the differences of the effect of 3 min vs. 30 s pre-incubation refer the symbols: n.s.; ## *p* < 0.01; ### *p* < 0.001. The statistical test used in order to assess significance of differences was Student’s t test. The *insert* illustrates, as an example, a single experiment with preincubation with AF-16, 0.01 µM for 3 min (trace in the center). The trace to the left refers to the control (plain GABA 10 µM ) and that to the right refers to the re-application of GABA 10 µM after washing out AF-16. (Reproduced with permission, license Springer Nature number: 6000160856523)

**Fig. 4:**
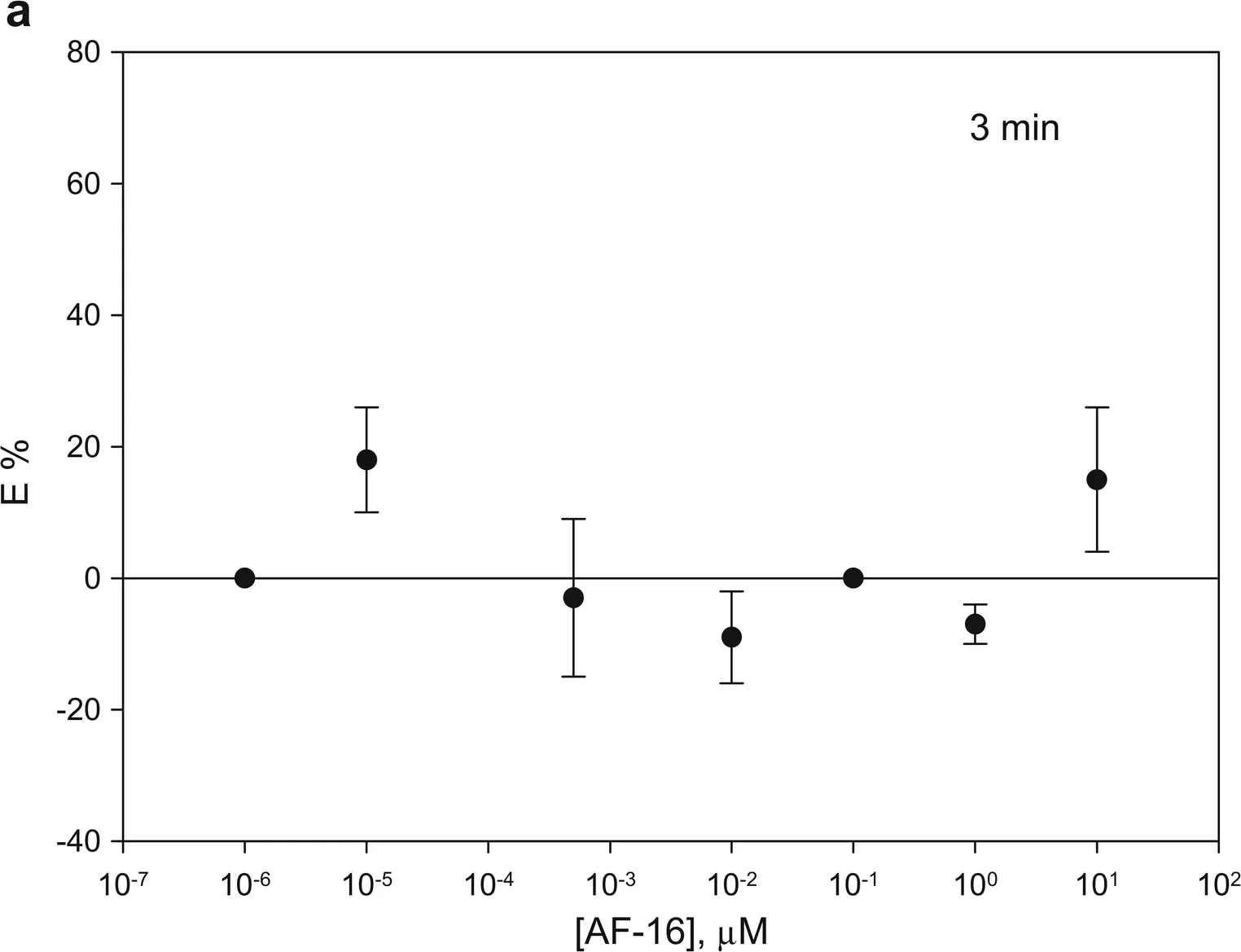
Evidence of the absence of stimulation of the *steady state chloride currents* activated by 10 µM GABA after 3 min perfusion of increasing concentrations of AF-16. The number of experiments in each case were at least three. The vertical bars represent SEMs. The statistical test used in order to assess significance or not of differences was Student’s t test. (Reproduced with permission, license Springer Nature number: 6000160856523)

The model of cerebellar granule cells in primary cultures has been used to test the influence of AF on neuronal GABA_A_ receptors activity. In particular, we used the AF-16 peptide. Perfusion of cells for three minutes with a constant AF-16 concentration dose-dependently increased the peak component of the chloride current activated by 10 µM GABA (Fig. 3 and its insert). No effect was found for the steady state component (Fig. 4). The increase reached a plateau of around + 40%, at AF-16 concentrations of 0.01 µM and above (Bazzurro et al. 2018).

Our immunocytochemical studies about the expression of the γ_2_ subunit of the GABA_A_ receptor showed that the increase of the peak current achieved by 1 µM AF-16, was accompanied by an increased expression of that subunit on cell membrane (+ 17.4 ± 0.4%, *n* = 32, *p* < 0.001; Bazzurro et al. 2018). The increase in the level of cell membrane γ_2_ subunit is consistent with an increase of the peak current.

**Fig. 5:**
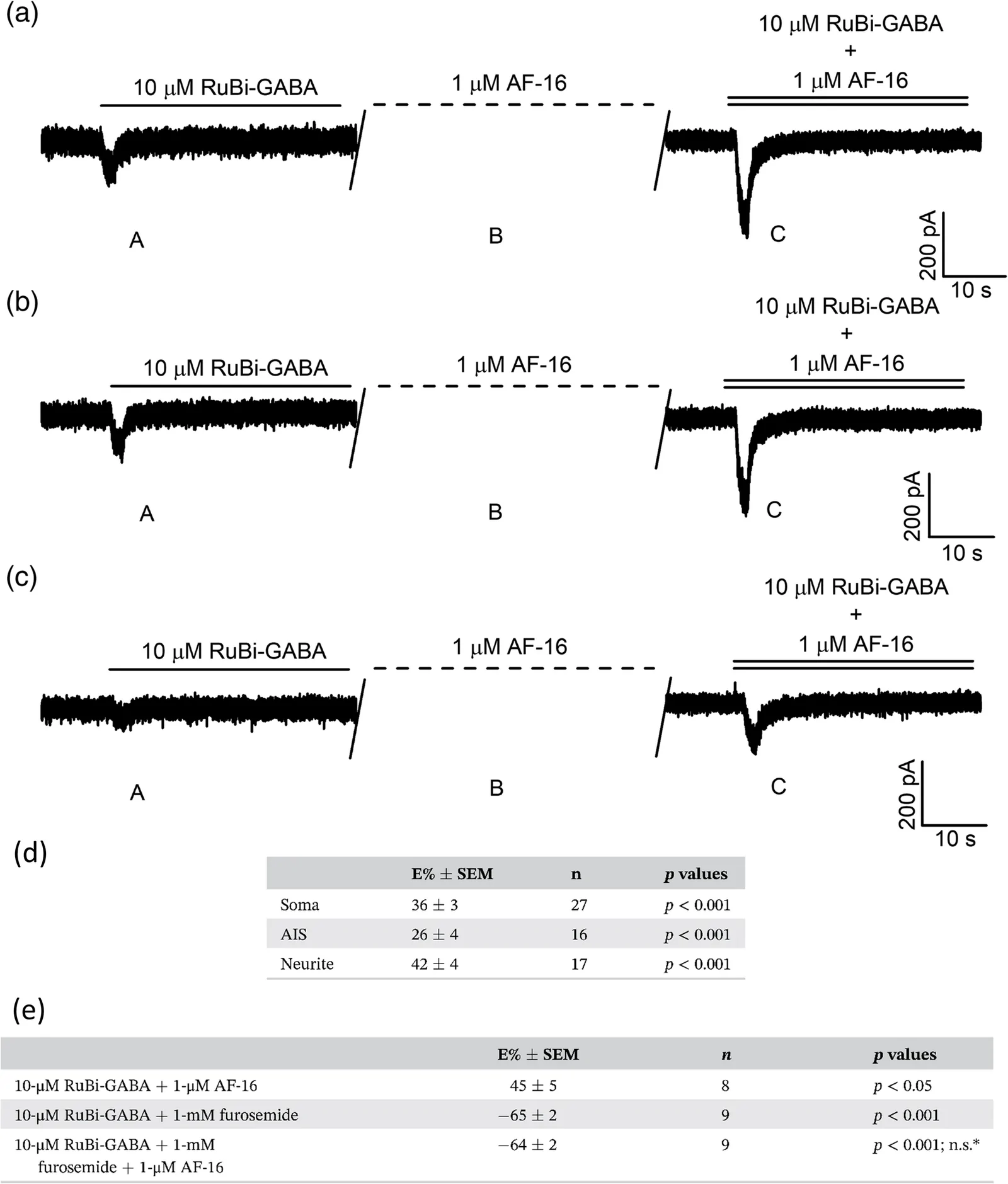
Examples of chloride currents activated locally by uncaging GABA from 10 µM RuBi-GABA: **(a)** soma; **(b)** axon initial segment (AIS); **(c)** neurites. Traces to the left represent control currents, effects of plain 10 µM RuBi-GABA. Traces to the right represent currents due to 10 µM RuBi-GABA after 3 min incubations with 1 µM AF-16; **(d)** percent (E %) increase of current after 3 min incubation with AF-16 by uncaging 10 µM RuBi-GABA on soma, axon initial segment (AIS) and neurites; Note: The data are given as mean ± SEM; n is the number of experiments, p values calculated with Student’s t test. Abbreviations: AF, antisecretory factor; AIS, axon initial segment. **(e)** percent effect (E%) of 1 µM AF-16 (first row), only 1 mM furosemide (second row) and 1 mM furosemide plus 1 µM AF-16 (third row) on 10 µM uncaged RuBi-GABA induced currents. The incubations with AF-16 were for 3 min. The E % were calculated in all the three cases in reference to the control (no AF-16, no furosemide). Note: For each data series, the number of experiments (n) and the statistical significance (p values) of the results are reported. The data are given as mean ± SEM; statistical comparisons were analysed with Student’s t test versus the control (no AF-16, no furosemide): p values. The last column reports the comparison between the 10-µM RuBi-GABA + 1-mM furosemide + 1-µM AF-16 and the 10-µM RuBi-GABA + 1-mM furosemide case (n.s.*: not significant). Abbreviation: AF, antisecretory factor. (Reproduced with permission, license John Wiley and Sons number: 6032950664174)

In additional experiments (Bazzurro et al. 2022) we applied the technique of uncaging “caged GABA”, RuBi GABA, by two-photon close to three different parts of the granule neurons: soma, axon initial segment (AIS) and neurites. The purpose was to test whether different parts of these neurons reacted differently to the same amount of GABA released from 10 µM RuBi-GABA. In all three cases, the current elicited by uncaging GABA was increased in the presence of 1 µM AF-16, however, the increase in the AIS was relatively lower (Fig. 5). The increase in both the cell soma and neurites was around 40%. It must be underlined that the effects we measured with the whole cell approach were mainly related to the cell body. From a quantitative point of view, the contribution by neurites was evidently low.

Further studies of the currents evoked by caged GABA on the cell soma in the presence or absence of 1 µM AF-16 were performed in the presence of furosemide, a blocker of GABA_A_ receptors containing the α6 subunit (Korpi and Luddens 1997). The results are reported in Fig. 5 and show that the percent decrease mediated by furosemide of the current in the respect of the basal condition (only GABA) is around 60% both in the absence and presence of AF. This demonstrates that the extra current elicited by AF-16 is entirely due to the α6 subunit containing GABA receptors.

Subsequently, we performed studies by immunofluorescence of α1 and α6 subunits in cerebellar neurons with and without 1 µM AF-16 treatment. The experimental conditions were followed by analyzing with super-resolving images. The quantitative presence of the two subunits in the cell bodies vs. neurites was evaluated. The results showed that the α1 subunit presented an increase in neurites and a decrease in the cell body, while the opposite result was found for the α6 subunit (Bazzurro et al. 2024).

## Discussion

Preincubation of cerebellar granule cells with 1 µM AF-16 increased the peak chloride current elicited by 10 µM GABA by approximately 40% (Bazzurro et al. 2018). The peak current increase appears to be the same in the cell body and in neurites, whereas it is lower in the axon initial segment (AIS) (Bazzurro et al. 2022). These increases involve mainly GABA_A_ receptors containing the α6 subunit, of the α_6_β_2/3_γ_2_ type (Robello et al. 1999; Gatta et al. 2009). Based on the amplitude of the GABA stimulatory effect, AF-16 does not display an increase of the steady state current (Bazzurro et al. 2018). It is useful to remember that the peak chloride current activated by GABA in our experimental setting corresponds to the synaptic fast inhibitory current activated by GABA at the Golgi cells – Granule neurons contacts, whereas the steady state one corresponds to the tonic one activated by sub-micromolar concentrations of extracellular GABA (Hamann et al. 2002; Farrant and Nusser 2005). In terms of the physiological effect of AF, once reaching an active concentration range in the tissue, it implies an increased GABA mediated inhibitory brake on cerebellar granule cells, resulting in less excitation of the Purkinje cells. These mediate the interaction of the cerebellum with other brain regions, e.g. via DCN. In particular, a diminished inhibitory brake on DCN and their connections with other brain structures, e.g. thalamus and the ventral tegmental area, VTA (Chiou and Sieghart 2025). In addition, the GABAergic Purkinje cells directly, mono-synaptically, inhibit the big Deiters’ neurons of the LVN in the brain stem (Ito and Yoshida 1966; Ito et al. 1966; Ito 2006; D’Angelo 2018). Whereas the interpretation of these results is relatively straightforward, it is more difficult to interpret the reciprocal changes between the α1 and α6 subunits within the cerebellar granule cells as being a result of the action of AF-16. In these cells, the two subunits redistribute with α6 decreasing in neurites and increasing in the cell body. The opposite reaction occurs to the α1 subunit (Bazzurro et al. 2024).

From our experiments it is not possible to discriminate which particular GABA_A_ receptors are arising in the cell body as a consequence of the increase of α6 receptors there, whether of the α_6_β_2/3_γ_2_ or of the α_6_β_2/3_δ type. However, our results via the whole cell approach suggest a specific increase of the peak current by AF-16 and indicate that this current increase is due to α_6_β_2/3_γ_2_ GABA_A_ receptors of the cell body. Although the whole cell approach also involves neurites, the great majority of the currents registered involves the soma of the cell. The synaptic fast chloride current is also due to receptors of the α_1_α_6_β_2/3_γ_2_ and α_1_β_2/3_γ_2_ types. However, those receptors are endowed with less affinity for GABA and more rapid rise and decay time than α_6_β_2/3_γ_2_ (Saxena and Macdonald 1996; Karim et al. 2013; Sieghart et al. 2022; Sieghart 2024), thus they have less impact on synaptic GABAergic inhibition via GABA_A_ receptors.

Overall, our in vitro results imply that the presence of a relevant concentration of AF in the extracellular space results in a more efficient inhibitory brake on the cerebellar granule cells with consequences for the circuitries where the cerebellum has a role. In particular, a decreased excitatory input to Purkinje cells, which are inhibitors of DCN neurons, will result in a greater activity of DCN neurons, one of the outputs of activity of cerebellar circuitry. Less active GABAergic Purkinje cells will, in turn, decrease their direct control of extra cerebellar nuclei such as the LVN. AF has been shown to influence synaptic activity and circuitry in the hippocampus. In particular, it reduces the inhibitory brake on CA1 pyramidal neurons with a consequent higher excitability of such neurons, this facilitates plastic changes, such as in vitro long term potentiation of the connection CA3-CA1 and in vivo formation of memory in the rat (Strandberg et al. 2014; Wintzell et al. 2022). Thus, it appears that there is a sort of parallelism between the final outcome of AF intervention on the hippocampal circuitry and on the cerebellar one. In both cases, the final outcome of its influence is to strengthen the activity of functionally pivotal neurons: CA1 pyramidal neurons in the hippocampus, whereas in the cerebellum on one hand DCN neurons, one of the main excitatory outputs, and on the other hand the vestibular Deiters’ neurons of the lateral vestibular nucleus in the brain stem, via decrease of Purkinje cell mediated inhibition.

It has been reported that Menière’s syndrome symptoms can be ameliorated by inducing AF production in the body by SPC-Flakes. In particular vertigo symptoms appear to be mitigated in these patients (Hanner et al. 2003, 2004, 2010; Leong et al. 2013; Scarpa et al. 2020; Viola et al. 2020). Our results indicate an augmented inhibitory brake on cerebellar granule cells by AF, the result of which is an increased excitatory output from cerebellar DCN (Sieghart 2024) and a diminished direct inhibitory brake by Purkinje cells on the big Deiters’ neurons of the LVN (Ito et al. 1966; Fenardjian and Sarkisian 1988; Hydén et al. 2000; Ito 2006). These neurons activate anti-gravity motor neurons of the spinal cord via the Lateral Vestibulo Spinal Tract which may help to counteract vertigo in Menière’s patients.

Clearly, ours is a mainly theoretical suggestion which we discuss and present in order to stimulate further studies testing whether our hypothesis is correct and worthwhile to pursue both at a scientific and, hopefully, a translational level. Direct studies in animal models of the AF effect on the electrophysiological activity of the neurons of interest in the control of vestibular neurons, in particular Purkinje cells, would give important answers.

## Final remarks

The present review paper describes the affected electrical activities of rat cerebellar granular cells in response to AF-16. The improved effect on the vertigo part of Menière’s disease in this context can probably be ascribed to a modified activity of the vestibulo-cerebellar system mediated via interaction with GABA_A_ receptors containing the α6 subunit.

Clinical studies have demonstrated that AF therapy is beneficial in diseases where secretion and/or inflammation dominate the patient’s symptoms, e.g. diarrheal diseases (Bjorck et al. 2000; Eriksson et al. 2003; Laurenius et al. 2003; Zaman et al. 2014, 2018), Menière’s disease (Hanner et al. 2003, 2004, 2010; Leong et al. 2013; Scarpa et al. 2020; Viola et al. 2020), mastitis (Svensson et al. 2004) and a traumatically induced increase of ICP due to brain edema (Gatzinsky et al. 2020). Further studies are required to investigate whether interaction with GABA_A_ receptors can explain the positive, regulating effects of AF in these pathophysiological conditions.

Sophisticated and detailed studies must be designed and performed in vivo and in vitro in order to achieve a more detailed understanding of the AF mode of action in the diseased and in the normal tissue, respectively. In the experimental models to be envisaged, we can primarily focus on measuring water and ion transport across cellular membranes. The results might hopefully provide us with data explaining AF´s effects in health and disease. In a further perspective this type of data can supply information that will enable the introduction of new therapeutic forms for treatment of diseases in which the cellular membranes are pathologically affected.

## Data Availability

No datasets were generated or analysed during the current study.

## Abbreviations

AF: Antisecretory Factor
AF-16: a peptide, comprising 16 aa, derived from the amino terminal end of AF protein
AIS: Axonal Initial Segment
DCN: Deep Cerebellar Nuclei
ICP: IntraCranial Pressure
LVN: Lateral Vestibular Nucleus
SPC: Specially Processed Cereals
VTA: Ventral Tegmental Area
